# Air quality prediction and control systems using machine learning and adaptive neuro-fuzzy inference system

**DOI:** 10.1016/j.heliyon.2024.e39783

**Published:** 2024-10-25

**Authors:** Pouya Mottahedin, Benyamin Chahkandi, Reza Moezzi, Amir M. Fathollahi-Fard, Mojtaba Ghandali, Mohammad Gheibi

**Affiliations:** a Department of Chemical Engineering, Faculty of Engineering, University of Garmsar, Garmsar, Iran; b Faculty of Civil and Environmental Engineering, Gdansk University of Technology, Narutowicza Street 11/12, 80-233, Gdansk, Poland; c Faculty of Mechatronics, Informatics and Interdisciplinary Studies, Technical University of Liberec, 461 17, Liberec, Czech Republic; d Association of Talent under Liberty in Technology (TULTECH), Sopruse Pst, 10615, Tallinn, Estonia; e Département d′Analytique, Opérations et Technologies de l′Information, Université du Québec à Montréal, B.P. 8888, Succ. Centre-ville, Montréal, QC, H3C 3P8, Canada; f Institute for Nanomaterials, Advanced Technologies and Innovation, Technical University of Liberec, 461 17, Liberec, Czech Republic; g Environment Research Center, Department of Environment, Semnan University, Semnan, Iran

**Keywords:** Air quality, Artificial intelligence, Machine learning, Adaptive neuro-fuzzy inference system

## Abstract

Accurately predicting air quality concentrations is a challenging task due to the complex interactions of pollutants and their reliance on nonlinear processes. This study introduces an innovative approach in environmental engineering, employing artificial intelligence techniques to forecast air quality in Semnan, Iran. Comprehensive data on seven different pollutants was initially collected and analyzed. Then, several machine learning (ML) models were rigorously evaluated for their performance, and a detailed analysis was conducted. By incorporating these advanced technologies, the study aims to create a reliable framework for air quality prediction, with a particular focus on the case study in Iran. The results indicated that the adaptive neuro-fuzzy inference system (ANFIS) was the most effective method for predicting air quality across different seasons, showing high reliability across all datasets.

## Highlights

• Introduces a new method for air quality prediction using machine learning and ANFIS.
• Targets air quality forecasting for Semnan, Iran.
• Utilizes comprehensive datasets and ten machine learning methods.
• Highlights ANFIS's reliability across seasonal variations.
• Establishes a basis for effective air quality control strategies.

## 1 Introduction

Rapid urbanization, industrial growth, and rising transportation demands have exacerbated air pollution, especially in densely populated cities [1,2]. This has led to significant health concerns, including eye disorders, fatty liver disease, mental health issues, and respiratory and cardiovascular conditions [[3], [4], [5], [6]]. The World Health Organization reports that 90 % of the global population breathes air exceeding quality standards, underscoring the urgency of the issue [7]. Accurate air quality prediction is crucial for safeguarding public health by enabling timely alerts about hazardous pollution events [[8], [9], [10], [11]].

Air pollutants are categorized into primary and secondary pollutants, such as SO₂, NO₂, CO, O₃, and particulate matter, which are responsible for numerous health issues [[12], [13], [14]]. National monitoring organizations measure these pollutants to prevent health complications, and predictive systems based on pollutant levels enhance the comprehensibility of air quality measurements, allowing for public health promotion [14,15]. Elevated pollution levels trigger health alerts, providing regulators with lead time to implement emission reduction and other responses. Monitoring station data is crucial for assessing air quality but is limited in time and space [16,17]. Prediction models help overcome these limitations, offering insights for managing emissions and decision-making during high-pollution events. Despite advancements, the complexity of air pollution dynamics presents challenges for accurate prediction, necessitating novel methodologies to enhance reliability [18,19].

Extensive research on predicting air quality highlights two main categories of models: mechanistic models and statistical models [13,20]. Mechanistic models use mathematical methods combined with meteorological parameters to explain complex atmospheric processes, though they often struggle to accurately detect air pollution concentrations [21]. In contrast, statistical models, such as those employing artificial intelligence, analyze pollutant data and predict concentrations over time, simplifying calculations and offering independence from specific physical mechanisms [[22], [23], [24]].

Statistical prediction methods widely use artificial neural networks (ANN) and machine learning (ML) techniques, which excel at identifying nonlinear relationships and often outperform conventional approaches [[25], [26], [27], [28], [29]]. Common ML models include support vector regression [30], multiple linear regression [31], random forest [32], and Gaussian process regression. ANN-based approaches are efficient for air pollution prediction, capturing intricate nonlinear relationships [33]. ANN architectures used for forecasting include Bayesian neural networks [34], neuro-fuzzy neural networks [35], backpropagation neural networks [36], general regression neural networks [37], multilayer perceptrons [38], and the adaptive neuro-fuzzy inference system (ANFIS), which enhances speed and adaptability [39,40].

The ANFIS methodology has been applied in various studies, such as modeling SO₂ concentrations in Bor, Serbia [41], and predicting air temperature in Karnataka, India [42]. Other studies include the short-term prediction of PM₁₀ in Romania [43], forecasting PM₂.₅ in Tehran [44], and predicting CO concentrations in Tabriz [45]. Hybrid approaches combining ANFIS with other methods have also been used to evaluate air quality in Jeddah [46] and analyze soft computing applications in air quality modeling [47]. Additionally, ANFIS has been employed to simulate air quality indices in India [48], assess traffic noise pollution in Mashhad, Iran [49], and estimate NOₓ emissions from power plants [50].

In order to forecast PM_2.5_ in Hefei, Yu et al. [51] created a spatiotemporal model that used spatial weighting, empirical mode decomposition (EMD), and long short-term memory (LSTM). Weights were assigned using LSTM to capture nonlinear features, Pearson correlation, and EMD for denoising. Ten monitoring stations' worth of data were used to predict next-hour concentrations. Fan et al. [52] used zebrafish embryos exposed to PM_2.5_ from Harbin and Hangzhou to study PM_2.5_ toxicity. Organ abnormality was discovered through microscopic inspection. Four predictive algorithms were used; random forest showed the strongest connections with PM_2.5_ components. Mohammadi et al. [53] used different models to predict PM_2.5_ in Isfahan. Using ArcGIS IDW (Inverse Distance Weighting) to visualize nine years' worth of weather data from seven stations. ANN demonstrated potential for managing air pollution by outperforming other algorithms in terms of accuracy. Application-Strategy-based LSTM (ASLSTM) was proposed by Lin et al. [54] to forecast PM_2.5_ hourly concentrations in Taichung. For spatiotemporal characteristics, Better LSTM (BLSTM) was trained using historical data. In order to improve next-hour PM_2.5_ estimates, ASLSTM iteratively integrated BLSTM outputs, demonstrating accuracy for high-concentration occurrences.

Despite various attempts at modeling and controlling air quality in different regions, the unbalanced development trajectory of Semnan province in Iran, marked by the concentration of industrial centers, has contributed to a troubling escalation in air pollution levels. Recent surveys underscore the operation of approximately 517 industrial units within the relatively confined area of Semnan, intensifying the challenges associated with combating air pollution. Consequently, conducting a comprehensive assessment of air pollution in Semnan has emerged as an imperative necessity, highlighting the critical significance of the present study. Hence, this research is structured to achieve the following key objectives.

• Evaluate the effectiveness and reliability of diverse machine learning methods for air quality prediction, with a specific focus on the assessment of the ANFIS.
• Undertake a comparative analysis of the results generated by the aforementioned algorithms, providing insights into the most accurate and efficient models based on five essential factors.
• Compute the correlation coefficients of various prediction methods using an extensive 18-month dataset, meticulously classified according to seasonal variations.
• Develop an optimized soft sensor for controlling air quality in densely populated urban centers, presenting a potential solution to the persistent challenges of air pollution.

By accomplishing these objectives, this research endeavors to establish a robust framework for effective air quality management, paving the way for sustainable environmental practices in Semnan and potentially serving as a guiding model for regions worldwide grappling with similar environmental challenges.

The subsequent sections of this paper are structured as follows: In Section 2, we elucidate the materials and methods employed in the prediction and management of air quality in Semnan, Iran. Our computational analyses and discussions are presented in Section 3. Finally, Section 4 concludes this study, emphasizing key limitations, and recommendations, and offering suggestions for future research avenues.

## 2 Materials and methods

This section outlines the materials and methods used in the study. Section 2.1 presents the research roadmap. Section 2.2 discusses the case study. Section 2.3 covers data acquisition. In Section 2.4, we describe the Random Forest (RF) model, followed by the M5P model in Section 2.5. Section 2.6 introduces the Random Tree (RT) model, while Section 2.7 explains the Decision Stump (DS) used in this study's machine learning methods. Section 2.8 focuses on the multilayer perceptron, and Section 2.9 discusses the Gaussian Process (GP) employed in the computations. Section 2.10 covers the Linear Regression (LR) model. Section 2.11 introduces the proposed ANFIS model, and finally, Section 2.12 presents the evaluation metrics used to assess these methods.

### 2.1 Research roadmap

This research evaluates the prediction efficiency of several prominent machine learning approaches alongside ANFIS, with the goal of methodically comparing their precision and effectiveness. The machine learning algorithms analyzed include Random Tree (RT), RF, M5P, DS, MLP, GP, LR, Simple Linear Regression (SLR), and SMOreg, with comprehensive details available in standard machine learning textbooks [28]. The prediction models are trained using historical data from air pollution monitoring stations in Semnan, providing valuable predictive insights. All machine learning models were simulated using WEKA 3.0 software, enabling a comprehensive comparative analysis of the results.

**Fig. 1:**
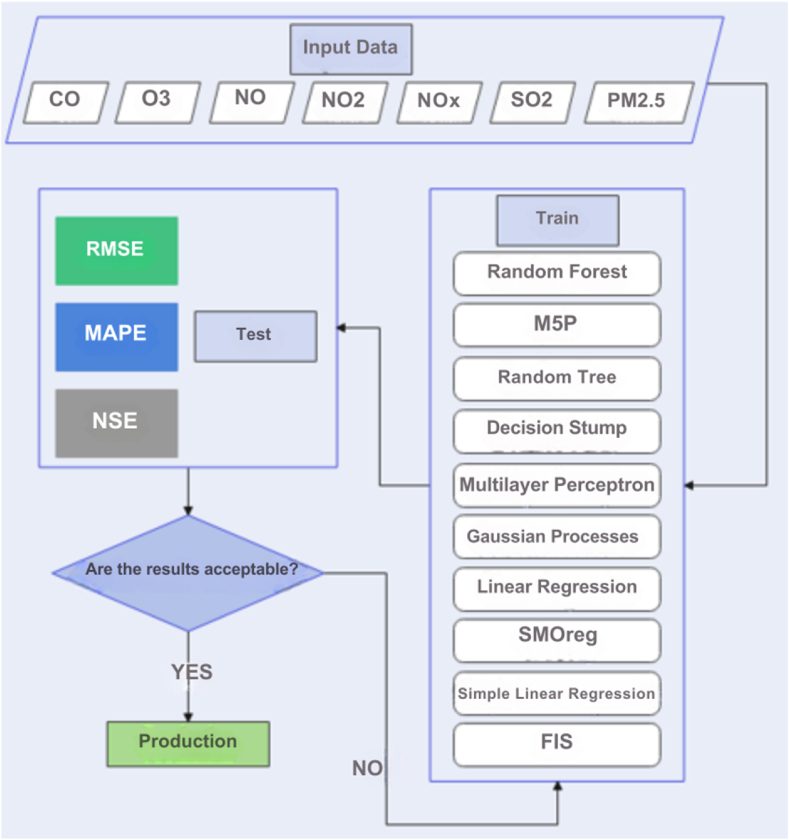
Research roadmap.

In parallel, the ANFIS methodology was implemented in MATLAB 2018b, configured with the Sugeno method, incorporating three triangle-type membership functions for each input feature. Additionally, the prediction optimization process was executed through a hybrid model over 35 epochs, ensuring thorough exploration of predictive capabilities. The initial dataset spans from 2020 to 2021, serving as the foundation for the analyses conducted in this study. Fig. 1 provides an illustration of the research roadmap, condensing the methodology and analytical approach adopted.

### 2.2 Case study

**Fig. 2:**
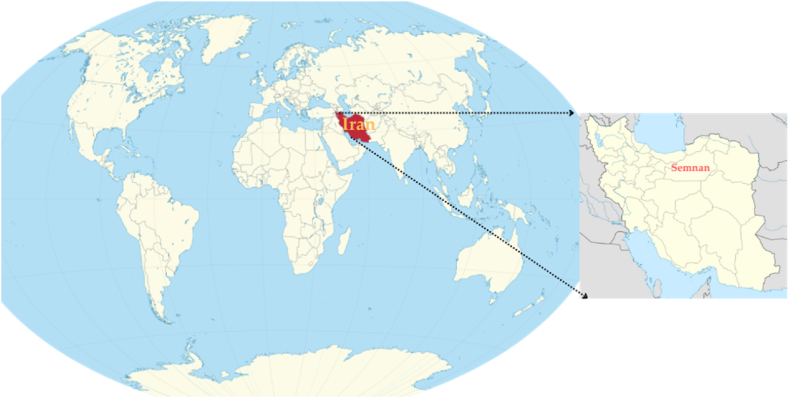
Semnan location in Iran as our case study.

Located in north-central Iran, Semnan is the capital of Semnan Province, with a population of approximately 185,129 residents. The city lies at an elevation of 1138 m above sea level, at the intersection of the Alborz Mountains' foothills and the expansive desert plains to the south. With the rainy season from December to May, Semnan experiences relatively light precipitation, resulting in a climate marked by intermittent cold, brisk winds that contribute to the city's chill factor. According to the Iranian Meteorological Department, Semnan has an average of 48 days per year with temperatures below freezing, highlighting the region's climatic characteristics. Fig. 2 shows Semnan's location within the landscape of Iran.

### 2.3 Data acquisition

This study used data from the Semnan city data collection, encompassing seven variables: NOₓ, CO, NO, O₃, NO₂, SO₂, and PM₂.₅. All data were sourced from the Semnan Environmental Protection Organization, ensuring accuracy and reliability. The dataset underwent thorough verification and correction, with a scheduled quarterly evaluation for ongoing accuracy. Table 1 provides an overview of the parameters, and their respective units used in the simulations.

**Table 1:** Model parameters.

Parameters	NO_x_	CO	NO	O_3_	NO_2_	SO_2_
**Unit**	ppb	ppm	ppb	ppb	ppb	ppb

The research provides an overview of the pollution data recorded in different seasons from the Semnan city, including a detailed descriptive analysis for each data sheet. The dataset contains information gathered over multiple seasons, spanning from Winter 2020 to Spring 2021.

The applied data library file consists of six sheets representing different seasons. The first-time section, Spring 2021, contains 142 rows and 7 columns (six inputs and PM2.5 as output). The Winter 2020–2021 time period contains 151 rows and 7 columns (six inputs and PM2.5 as output). The Autumn 2020 sheet includes 179 rows and 7 columns, with data covering 12-h readings. The Summer 2020 sheet records 114 rows and 7 columns of data, primarily focusing on summer pollution measurements. The Spring 2020 sheet contains 121 rows and 7 columns, with pollution readings primarily showing time-based information. The Winter 2020 sheet includes 69 rows and 7 columns, with data primarily focusing on time-based pollution measurements. Similar descriptive analysis was conducted for pollutant levels, giving an overview of pollution trends during this winter. The dataset provides 12-h pollutant measurements, enabling the study of daily trends and seasonal variations. Standard deviation values were analyzed to understand the variability in pollution levels between different seasons. Additionally, outlier detection was performed, highlighting extreme pollution episodes that may require further investigation. All initial computations are done in excel 2016 software and based on descriptive statistical analysis.

### 2.4 Random forest

Random Forest (RF) is a supervised machine learning technique that uses multiple decision trees to handle both regression and classification tasks. The RF algorithm generates several decision trees from different subsets of the training data, and each tree produces a class prediction. The final prediction is based on the most frequently occurring class for classification problems or the average of all tree responses for regression tasks. The Random Forest method follows four main steps: first, it selects random samples from the dataset; second, distinct decision trees are constructed for each sample; third, the results from the individual trees are aggregated; and fourth, the final result is derived based on the most-voted outcome for classification or by averaging for regression [55].

### 2.5 M5P

The M5P model is a type of decision tree designed for solving regression problems by combining decision tree and linear regression approaches. It uses an inductive algorithm to create the tree, where instead of maximizing data at each internal node, a split condition is applied to minimize intra-subset differences in class values [56]. The variability is measured based on the normal variation of data reaching each node, and the splitting process uses a standard deviation reduction (SDR) parameter to maximize the anticipated error decrease at every node. The splitting stops when class values differ very little or when there are too few samples at a node. This allows M5P to effectively address regression problems by leveraging both decision tree structure and linear regression for prediction.

(1) $$SDR=sd\left(S\right)-\sum_{i}\frac{S_{i}}{\left|S\right|}\times sd\left(S_{i}\right)$$

In M5P, *sd* represents the standard deviation, *S* is the dataset at a node, and *S*_*i*_ are the subsets obtained after splitting [56]. Once the tree is created, linear regression models are developed for each sub-space. Pruning starts at the leaves to prevent overfitting, converting internal nodes to leaves when error reduction is minimal. This pruning can create gaps between linear models of neighboring nodes, which are smoothed by combining predicted values along the path back to the root [[57], [58], [59]].

(2) $$E^{\prime }=\frac{ne+ka}{n+k}$$

Where $E^{\prime }$ is the approximated value went through the next higher node, *e* is the approximated value came to the present node from lower nodes, *a* is the forecasted value at the present node, *n* is the number of training instances that reach the lower node, at the end, *k* is a constant.

### 2.6 Random Tree

Random Trees, used as an ensemble of tree predictors, can handle both classification and regression tasks effectively [25]. For classification, each tree in the ensemble assigns a label to the input data, with the final label determined by the majority vote. For regression, the output is the average of the predictions from all the trees. Random Trees use ensemble learning, generating individual learners through the concept of bagging, which helps improve robustness across different scenarios. The trees are constructed by selecting the optimal split from all parameters at each node, while in a random forest, nodes are split using the best subset of randomly selected predictors, which enhances model diversity and resilience [[60], [61], [62]].

### 2.7 Decision stump

One of the fundamental techniques extensively utilized in the machine learning is the decision stump, operating as a one-level decision tree. This binary classification algorithm embodies a core principle of machine learning, emphasizing the singular focus on one specific feature at a time. It efficiently seeks an optimal point that effectively separates the data, as represented by Eq. (3).

$$H=\left\{h_{a,c}:a\in R,c\in +1,-1\right\}$$

(3) $$h_{\left(a,c\right)}\left(x\right)\left\{c=-1ifx\leq a,c=+1ifx>a\right\}$$

Number a should be found in a way to best classify the training data. As in reality finding such number that can perfectly separate the data is mostly impossible, a way to measure the performance has to be initialized. One of the best solutions to achieve this is the Gini index as defined in Eq. (4) which can evaluate the purity of the two subgroups. If Gini index is equal to zero, two subgroups are entirely pure.

(4) $$G_{i}=1-{\sum_{k=1}^{K}\left(\frac{\sum_{n}^{k}\left[y_{n}=k\right]}{N}\right)}^{2}$$

where *k* represents different categories.

### 2.8 Multilayer perceptron

A Multilayer Perceptron (MLP), a type of feedforward ANN, exhibits a remarkable capability to construct intricate nonlinear models, rendering it particularly suitable for the task of air quality forecasting. The MLP typically consists of at least three layers: an input layer, a hidden layer, and an output layer. Each node, except for the input nodes, functions like a neuron, using nonlinear activation functions to process the information. Distinguished from a linear perceptron by its multiple layers and nonlinear activation function, the MLP stands as a versatile tool for complex data analysis [[63], [64], [65]].

In operation, the MLP commences when input values are introduced into the network. These input values generate signals that propagate through the network, beginning at the input layer, passing through the hidden layer, and ultimately reaching the output layer. Real numerical quantities, known as weights, are multiplied by the scaled input vector provided by the neurons in the input layer. Moreover, the neuron in the hidden layer incorporates the previously mentioned information summarized as bias (*b*), as indicated in Eq. (5).

(5) $$y_{0}=\sum_{i=1}^{n}w_{i}x_{i}+b$$

In this stage the weighted sum of the information is still in the linear format. When the information passes through the transfer function, the non-linearity takes place as Eq. (6).

$$f\left(x\right)=\frac{1}{1+e^{-x}}$$

(6) $$y_{0}=f\left(x\right)\left[\sum_{i=1}^{n}w_{i}x_{i}+b\right]$$

where *y*_*0*_ is the result, *w*_*i*_ and *x*_*i*_ represent weight vector and scaled feeding vector, *b* refers to bias, *x* is the total number of weighted inputs, and *f* is the transfer function.

Upon the computation of the error signal, the algorithm ceases the model fitting process. The error signal in the simulation, corresponding to the input, is computed as the variance between the desired outcome and the obtained results. The MLP, characterized by several neurons, is articulated in Eq. (7) as follows.

(7) $$y_{0}=f\left[\sum WO_{kj}(\sum_{i=1}^{n}WI_{ij}x_{i}+b_{1})+b_{2}\right]$$

where *WO*_*kj*_ is weight of output layer, *WI*_*ij*_ is the weight of input layer, the bias in the input layer is *b*_*1*_, while the bias in the output layer is *b*_*2*_.

### 2.9 Gaussian Processes

Gaussian Processes (GP) extend the Gaussian probability distribution to create non-parametric machine learning methods suitable for both classification and regression tasks. As a kernel model, they enable precise prediction of class membership probabilities. However, selecting and configuring the appropriate kernel can be challenging [66,67]. This method operates on the premise that the training dataset comprises an input variable matrix, *X*, and an output variable matrix, *Y*. Leveraging Gaussian Process Regression (GPR), it establishes a nonlinear relationship between *X* and *Y*, as illustrated in Eq. (8).

(8) Y=f(X)+ε

where the Gaussian noise is ε. If the data are scaled correctly, it is assumed that the regression function already has a zero mean Gaussian distribution, as shown in Eq. (9).

(9) $$Y=\left[f\left(x_{1}\right),f\left(x_{2}\right),\ldots ,f\left(x_{n}\right)\right]∼GP\left(0,C\right)$$

*C* is an n×n covariance matrix. The squared exponential covariance function as Eq. (10), is one of the frequently used covariance functions.

(10) $$C\left(x_{i},x_{j}\right)={\sigma_{f}}^{2}\;\exp\left\{-\frac{1}{2}{\left(x_{i}-x_{j}\right)}^{T}M^{-1}\left(x_{i}-x_{j}\right)\right\}+\delta_{ij}{\sigma_{n}}^{2}$$

If *i=j*, then $\delta_{ij}=1$, otherwise it is equal to zero, *M=l*^*2*^*I* where *l* is a parameter to scale length and determine the softness of the model, also *I* indicate an identify matrix and $\sigma_{f}^{2}$ resembles the signal variance. A log-likelihood function as below should be maximized to determine the hyper-parameter $\theta \left(l,\sigma_{f},\sigma_{n}\right)$ of the GPR model in Eq. (11).

(11) $$L=-\frac{n}{2}\log\left(2\pi \right)-\frac{1}{2}\log\left(\det\left(C\right)\right)-\frac{1}{2}Y^{T}C^{-1}Y$$

The covariance function in Gaussian Processes can be any function that generates a non-negative definite covariance matrix. In addition to the commonly used squared exponential covariance function, other options like linear, constant, rational quadratic, polynomial, periodic, and neural network functions can be utilized. Composite covariance functions can also be used to create new kernels from existing ones. In the current study, three fundamental kernels, i.e., linear, squared exponential, and periodic, have been selected for analysis.

### 2.10 Linear regression

Linear regression is a fundamental tool for predicting continuous variables, highlighting the linear relationship between dependent and independent factors. It captures how changes in the independent variables affect the dependent variable. There are two main types: SLR, which uses one independent variable, and Multiple Linear Regression, which uses multiple independent variables for prediction [45,[68], [69], [70], [71], [72]]. Single LR establishes a relationship between a simple predictor variable and a singular independent or response value. The comprehensive specification of SLR is illustrated in Eq. (12).

(12) Y=α+βx+ε

where *α* is a constant number usually defined as the intercept it tells the value of the response when the slope is equal to zero. *β* is another constant called coefficient representing the slope of the regression line and ε is a random parameter regarded with a mean of 0 and a variance of *σ*^*2*^ and also possess a bell-shaped or Gaussian distribution. In a given set of data, SLR finds these parameters by minimizing the squared distances between the line and the data points.

### 2.11 Adaptive neuro-fuzzy inference system

**Fig. 3:**
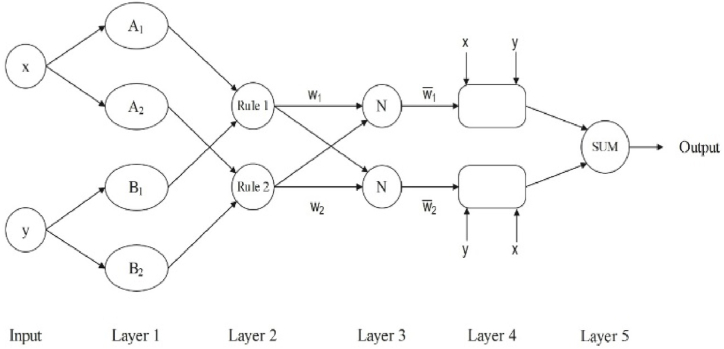
Overview of the applied ANFIS method.

ANFIS is a hybrid model that integrates least-squares and backpropagation gradient descent to train Takagi-Sugeno-type fuzzy inference systems, helping to identify optimal parameters. It uses fuzzy "if-then" rules to establish input-output relationships. ANFIS has a simple structure with an efficient learning algorithm, benefiting from faster convergence due to the backpropagation mechanism, which reduces the search space dimensions as approved in Fig. 3 [73,74].

The overview of ANFIS is demonstrated in Fig. 3 in which the inputs of layer 1 are x and y; the ${O_{i}}^{1}$ denotes the output of the node *i*. The brief procedure of this algorithm is described in Eq. (13).

$${O_{i}}^{1}=\mu_{A_{i}}\left(x\right),\forall i\in \left\{1,2\right\}$$

$${O_{i}}^{1}=\mu_{B_{i-2}}\left(y\right),\forall i\in \left\{3,4\right\}$$

(13) $$\mu \left(x\right)=e^{-{\left(\frac{x-\rho_{i}}{\alpha_{i}}\right)}^{2}}$$

where ${O_{i}}^{1}$ is the *i*th output of the layer *1*, *A*_*i*_ and *B*_*i*_ display the membership values of *μ*, which *μ* is the generalized gaussian membership function. *α*_*i*_ and *ρ*_*i*_ represent the supposed parameter set. In the *2nd* and *3rd* layers the process is as Eq. (14).

$${O_{i}}^{2}=\mu_{A_{i}}\left(x\right)\times \mu_{B_{i-2}}\left(y\right)$$

(14) $${O_{i}}^{3}={\bar{w}}_{i}=\frac{\omega_{i}}{\sum_{i=1}^{2}\omega_{i}}$$

where *w*_*i*_ represents the *i*th output from the second layer.

In the *4*th layer, the result of the *3*rd layer is multiplied with the Sugeno fuzzy rule’s function as represented in Eq. (15).

(15) $${O_{i}}^{4}={\bar{w}}_{i}f_{i}={\bar{w}}_{i}\left(p_{i}x+q_{i}x+r_{i}\right);\forall i\in \left\{1,2\right\}$$

In which *p*_*i*_, *q*_*i*_ and *r*_*i*_ is the resulting parameters of the node *i*.

The weighted average summation method is employed in the fifth layer to calculate the final output as Eq. (16).

(16) $${O_{i}}^{5}=\frac{\sum_{i}\omega_{i}f_{i}}{\sum_{i}\omega_{i}}$$

### 2.12 Performance evaluation

In this research, the effectiveness of the applied methodologies was assessed using five metrics, including Root Mean Squared Error (RMSE), Mean Absolute Error (MAE), Relative Mean Absolute Error (RMAE), Root Relative Mean Squared Error (RRMSE), and correlation coefficient of determination (R^2^).

• Root mean squared error (RMSE):

(17) $$RMSE=\sqrt{\frac{1}{m}\sum_{i=1}^{m}{\left(x_{i}-{\hat{x}}_{i}\right)}^{2}}$$

• Mean absolute error (MAE):

(18) $$MAE=\frac{1}{m}\sum_{i=1}^{m}\left|x_{i}-{\hat{x}}_{i}\right|$$

• Relative mean absolute error (RMAE):

(19) $$RMAE=\frac{1}{m}\;\sum_{i=1}^{m}\left|\frac{x_{i}-{\hat{x}}_{i}}{x_{i}.{\hat{x}}_{i}}\right|$$

• Root relative mean squared error:

(20) $$RRMSE=\sqrt{\frac{\sum_{i=1}^{m}{\left(x_{i}-{\hat{x}}_{i}\right)}^{2}}{m\sum_{i=1}^{m}{\left({\hat{x}}_{i}\right)}^{2}}}$$

where *m* indicates the number of observations, $x_{i}$ is the real value and ${\hat{x}}_{i}$ is the forecasted value.

• Sample correlation coefficient of determination:

(21) $$R^{2}={\left[\frac{\sum_{j=1}^{m}\left[\left(y_{j}-\hat{y}\right)\left(x_{j}-\hat{x}\right)\right]}{\sigma_{y}\sigma_{x}}\right]}^{2}$$

Where m is the number of data points, *y*_*j*_ is the calculated values, *x*_*j*_ is the observed values, ${\;\sigma }_{x}and\sigma_{y}$ are the standard deviation of the observation *x* and *y* respectively, $\bar{x}$ is the mean of observed values and $\bar{y}$ is the mean of computed values.

## 3 Results and discussion

The data utilized in this study is sourced from the air pollution monitoring station in Semnan. Acquired on a daily basis and segregated by distinct seasons, this dataset serves as the foundation for training our machine learning algorithms, enabling the precise forecasting of major air pollutants (e.g., NOx, CO, NO, O_3_, NO_2_, SO_2_, PM_2.5_) within the city. To showcase the efficacy and accuracy of the algorithms presented in the subsequent section, the results have been accurately compared using five distinct metrics, with the outcomes detailed in Table 2, Table 3, Table 4.

**Table 2:** Performance indicators of the implemented algorithms for Winter and Spring 2020.

MODEL	Winter 2020					Spring 2020				
	R^2^	MAE	RMSE	RMAE (%)	RRMSE (%)	R^2^	MAE	RMSE	RMAE (%)	RRMSE (%)
Random Tree	0.456	6.503	12.745	99.57	94.22	0.673	11.131	17.954	321.94	466.99
Random Forest	0.801	5.727	11.699	87.69	86.49	0.831	1.902	2.038	55.02	53.01
M5P	0.913	3.377	3.987	68.41	77.06	0.327	4.001	5.873	81.61	92.52
Decision Stump	0.991	5.159	5.244	104.5	101.37	0.631	2.254	2.812	56.32	63.38
Multilayer Perceptron	0.999	2.433	2.538	49.29	49.05	0.346	6.361	8.148	158.98	183.63
Gaussian Processes	0.711	3.822	4.525	94.49	98.24	0.473	3.187	3.902	77.48	86.19
Linear Regression	0.943	2.871	3.158	58.16	61.04	0.305	5.673	7.385	126.71	147.61
Simple Linear Regression	0.962	3.224	3.439	65.31	66.47	0.383	5.072	6.232	123.31	137.67
SMOreg	0.955	3.328	3.769	67.42	72.86	0.94	0.383	0.384	13.68	12.04
ANFIS	0.999	0.0162	0.0274	0.13	0.23	0.999	0.4091	0.7575	3.14	5.82

**Table 3:** Performance indicators of the implemented algorithms for Summer and autumn 2020.

MODEL	Summer 2020					Autumn 2020				
	R^2^	MAE	RMSE	RMAE (%)	RRMSE (%)	R^2^	MAE	RMSE	RMAE (%)	RRMSE (%)
Random Tree	0.577	3.019	3.845	75.6	83.27	0.756	2.482	3.156	74.3	79.46
Random Forest	0.717	2.544	2.943	63.92	63.67	0.805	2.625	3.072	68.06	67.08
M5P	0.182	3.618	5.118	89.76	107.19	0.67	3.166	3.709	82.1	81.01
Decision Stump	0.817	2.431	2.925	67.559	66.11	0.635	3.649	4.229	94.62	92.35
Multilayer Perceptron	0.913	1.8	2.031	55.17	53.69	0.562	3.15	4.308	98.91	94.08
Gaussian Processes	0.947	2.218	2.596	69.76	68.41	0.605	3.382	4.042	87.68	88.27
Linear Regression	0.115	4.046	10.448	90.52	99.89	0.663	3.114	3.701	80.75	80.81
Simple Linear Regression	0.88	2.679	3.182	74.51	71.93	0.485	3.693	4.264	95.74	93.11
SMOreg	0.894	1.212	1.808	38.12	47.67	0.623	3.049	3.602	79.06	78.66
ANFIS	0.999	0.1014	0.2233	1.01	2.23	0.892	0.7217	1.3927	6.89	13.29

**Table 4:** Performance indicators of the implemented algorithms for Winter and Spring 2021.

MODEL	Winter 2021					Spring 2021				
	R^2^	MAE	RMSE	RMAE (%)	RRMSE (%)	R^2^	MAE	RMSE	RMAE (%)	RRMSE (%)
Random Tree	0.457	6.503	12.745	99.57	94.22	0.324	6.411	9.034	121.45	124.01
Random Forest	0.801	5.727	11.699	87.69	86.49	0.583	2.831	3.501	88.83	86.71
M5P	0.912	3.372	3.987	68.41	77.06	0.649	2.227	3.026	71.65	77.03
Decision Stump	0.981	5.159	5.244	104.51	101.37	0.235	4.727	7.201	88.16	98.89
Multilayer Perceptron	0.99	2.433	2.538	49.29	49.05	0.628	3.082	4.081	99.18	103.87
Gaussian Processes	0.711	3.822	4.525	94.49	98.24	0.177	5.261	7.114	98.2	98.26
Linear Regression	0.943	2.871	3.158	58.162	61.04	0.342	4.453	6.881	83.06	94.51
Simple Linear Regression	0.962	3.224	3.439	65.31	66.47	0.376	3.398	4.312	97.85	98.61
SMOreg	0.955	3.328	3.769	67.42	72.86	0.353	3.036	4.879	96.31	119.65
ANFIS	0.981	0.418	0.9495	2.88	6.53	0.982	0.5206	1.0395	5.47	10.92

In Winter 2020 as reported in Table 2, ANFIS emerges as the best model with an R^2^ of 0.999, indicating a near-perfect fit. It also achieves the lowest errors among all metrics, with MAE of 0.0162, RMSE of 0.0274, RMAE of 0.13 %, and RRMSE of 0.23 %, making it the most reliable model for this season. MLP also performs well with an R^2^ value of 0.999, although its MAE (2.433) and RMSE (2.538) are much higher than those of ANFIS, indicating larger prediction errors. The passage also highlights that Decision Stump performed well during Winter 2020, but its metrics suggest otherwise, as it had lower accuracy overall. Random Tree, with the lowest R^2^ (0.456), and high MAE (6.503) and RMSE (12.745), is the least effective in capturing the variability in the data. SMOReg performs moderately well with an R^2^ of 0.955, and its MAE (3.328) and RMSE (3.769) are in a similar range as GP and LR.

During Spring 2020 as reported in Table 2, ANFIS continues to dominate, with an R^2^ of 0.999, MAE of 0.4091, and RMSE of 0.7575. Its relative metrics (RMAE of 3.14 % and RRMSE of 5.82 %) further confirm its consistent effectiveness. Random Forest shows a decent R^2^ value of 0.831, but its higher MAE (1.902) and RMSE (2.934) suggest larger errors compared to ANFIS. The passage notes that ANFIS significantly outperformed all other models, while Random Forest performed relatively well. Decision Stump significantly underperforms with the lowest R^2^ (0.107), and high error metrics (MAE of 6.361 and RMSE of 8.148) indicate poor predictability. GP and MLP have moderate R^2^ values of 0.473 and 0.346 respectively, but both exhibits substantially larger MAE and RMSE values compared to ANFIS.

In Summer 2020 as reported in Table 3, ANFIS maintains its dominance, with an R^2^ of 0.999 and low error values (MAE of 0.1014, RMSE of 0.2233), indicating stability across seasonal changes. The relatively low RMAE (1.10 %) and RRMSE (2.23 %) further reinforce its stability. SMOReg also shows reasonable performance with an R^2^ of 0.994, though it falls short compared to ANFIS in terms of error metrics, with MAE of 1.212 and RMSE of 1.808. The passage also mentions that MLP and Gaussian Process follow ANFIS in accuracy, which is consistent with their R^2^ values, although their errors are notably higher. Random Tree, with an R^2^ of 0.577 and high MAE (3.019) and RMSE (3.845), is among the least effective models for this season. Decision Stump remains weak, with an R^2^ of 0.317 and relatively high error metrics.

During Autumn 2020 as reported in Table 3, ANFIS continues to outperform other models, though its performance dips slightly, with an R^2^ of 0.892, MAE of 0.7217, and RMSE of 1.3927. This increase in errors suggests some seasonal influence on its performance. SMOReg shows reasonable results with an R^2^ of 0.623, but the error metrics (MAE of 3.049 and RMSE of 3.602) are much higher compared to ANFIS. Random Forest performs better in autumn, with an R^2^ of 0.805, although its MAE (2.625) and RMSE (3.072) are still significantly higher than ANFIS. The passage also mentions that Random Forest and M5P displayed better R^2^ values in autumn, while Random Tree and Random Forest excelled in terms of MAE and RMSE, further highlighting their varied performances.

In Winter 2021 as reported in Table 4, ANFIS maintains its high accuracy with an R^2^ of 0.981, MAE of 0.4185, and RMSE of 0.4959, slightly higher than Winter 2020 but still better than other models. Its RMAE (8.28 %) and RRMSE (6.53 %) demonstrate a slight decline, but the performance is still the best overall. Random Forest and SMOReg are again the next best contenders, but their R^2^ values of 0.801 and 0.955 respectively are unable to match ANFIS's level of fit and accuracy. Decision Stump lags behind, with high error metrics (MAE of 5.159, RMSE of 5.244), indicating its continued inadequacy for accurate predictions during this season.

During Spring 2021 as reported in Table 4, ANFIS continues its superior performance with an R^2^ of 0.982, MAE of 0.4036, and RMSE of 0.4365, indicating minimal errors. The RMAE (5.47 %) and RRMSE (10.92 %) further demonstrate its efficiency and accuracy. Random Tree, once again, is among the least effective models, with an R^2^ of 0.324 and large error metrics (MAE of 4.611 and RMSE of 9.034). Multilayer Perceptron and Gaussian Process achieve moderate R^2^ values of 0.527 and 0.557 respectively but still lag behind in accuracy compared to ANFIS. The performance of Linear Regression remains consistent with previous seasons, with moderate R^2^ and higher error metrics.

**Fig. 4:**
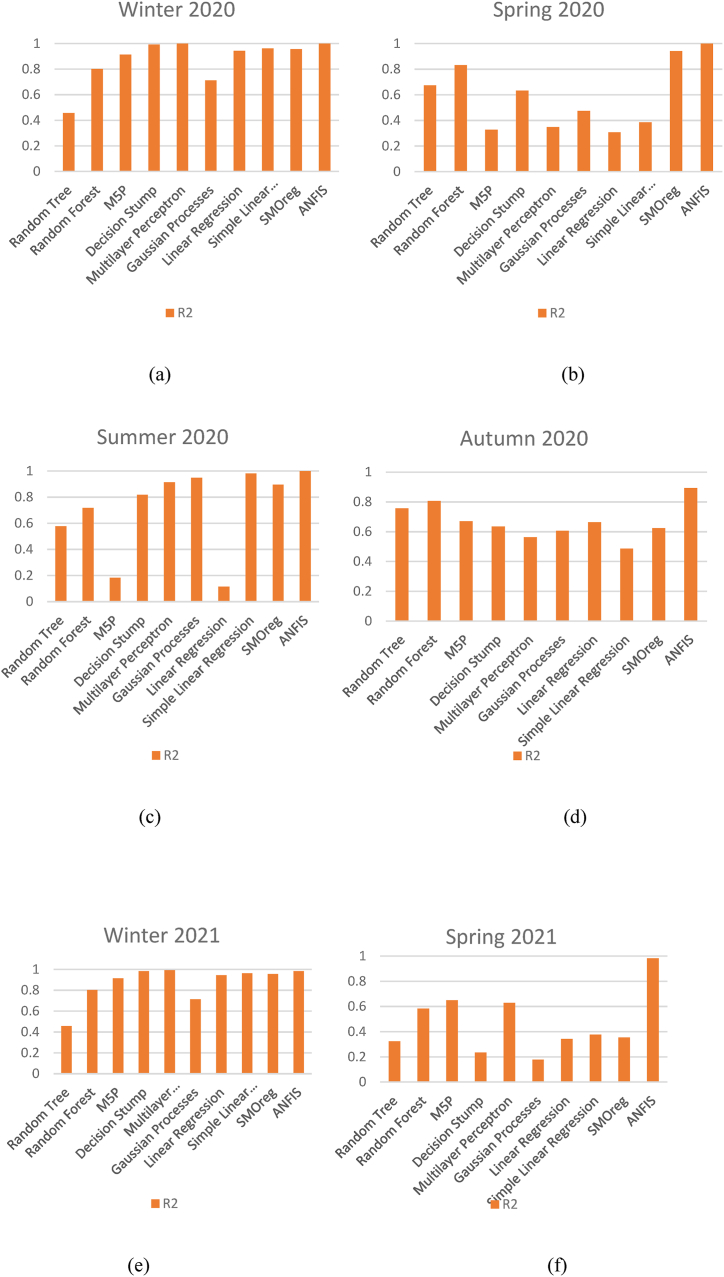
The comparison of all implemented algorithms in term of R^2^ a) winter 2020, b) spring 2020, c) summer 2020, d) autumn 2020, e) winter 2021, f) spring 2021.

In conclusion, ANFIS consistently outperforms all other models across all seasons, as evidenced in Table 2, Table 3, Table 4, and Fig. 4, which visually depicts comparative R^2^ performance. ANFIS demonstrates robustness and superior accuracy, with near-perfect R^2^ values and the lowest MAE, RMSE, RMAE, and RRMSE metrics across the seasons, making it the best choice for this domain. SMOReg and RF are consistently the next best performers, with reasonable R^2^ values and moderate error metrics, but still fall short compared to ANFIS. DS and SLR consistently underperform, exhibiting low R^2^ values and high errors, highlighting their unsuitability for complex seasonal prediction tasks. Additionally, the analysis shows that even ANFIS experiences slight seasonal variability, particularly in Autumn 2020, indicating that seasonal influences can affect model performance. Overall, ANFIS is the optimal choice across all seasons, offering high predictability and low error rates, while simpler models such as Decision Stump and Random Tree fail to deliver accurate predictions.

As a short comparison, Veljanovska & Dimoski [75] analyzed four learning algorithms, including the k-nearest neighbor (k-NN), Support Vector Machines (SVM), Decision Tree (DT), and Neural Network (NN) using datasets from Macedonia to predict air pollutant concentrations. They concluded that the NN method exhibited the highest efficiency, followed by SVM and KNN. Ameer et al. [76] conducted air pollution prediction in five cities in China, employing four regression methods, including Random Forest, Decision Tree, Multilayer Perceptron, and Gradient Boosting Regression, and evaluated their performance based on criteria such as MAE, RMSE, and processing time. They found that Random Forest demonstrated the overall best performance among all techniques, while Gradient Boosting Regression exhibited the worst performance in terms of error rate and processing time across most datasets. Taheri and Razban [77] employed six learning algorithms to investigate the forecasting potential of CO2 concentration, with the results indicating that MLP outperformed other algorithms in accurately forecasting CO_2_ behavior. Maleki et al. [78] used an artificial neural network (ANN) to predict the concentration of air pollutants and the air quality index in Ahvaz, one of the most polluted cities in Iran. They obtained a correlation coefficient of 0.87, demonstrating the applicability of ANN in predicting air quality for decision-makers. Nourani et al. [45] attempted to create a CO concentration prediction model in Iran, utilizing both ANN and ANFIS and achieving correlation coefficients of 0.82 and 0.63, respectively. Dirik [50] proposed a GA-ANFIS model to forecast NOx pollution from a biogas combined cycle power plant, achieving a correlation coefficient ranging from 0.79933 to 0.90363.

Fig. 4 presents a comparison of R^2^ values for various machine learning algorithms across different seasons from Winter 2020 to Spring 2021. Each subfigure (a to f) represents a distinct season, providing insights into the predictive performance of these algorithms. This type of analysis is critical from an industrial perspective, particularly in developing a real-time online dashboard or soft sensor for PM_2.5_ prediction in air quality monitoring applications.

In an industrial setting, specifically for air quality control and monitoring, the development of a real-time online dashboard is vital for predictive insights and timely decision-making regarding PM_2.5_ levels. A dashboard supported by machine learning-based soft sensors allows industries to predict PM_2.5_ concentrations, providing critical information to make operational decisions, such as adjusting emissions or activating filtration systems. Fig. 4 offers valuable insight into which algorithms might be best suited for such an application.

In Winter 2020 as shown in Fig. 4a, ANFIS, SMOReg, and Random Forest display the highest R^2^ values, close to 0.9 or above, indicating robust performance in predicting PM_2.5_ levels. In an online dashboard, using ANFIS as the underlying model for prediction would provide accurate real-time assessments of PM_2.5_, allowing timely interventions. Industrial operations with variable emissions, such as chemical plants or factories with seasonal output fluctuations, could rely on ANFIS for precise winter-time monitoring and control, as it appears to perform consistently well even in harsher, winter-specific conditions.

During Spring 2020 as shown in Fig. 4b, ANFIS again outperforms other algorithms, while Random Forest also shows promising performance, though with a slightly lower R^2^ value compared to ANFIS. The performance of models like Decision Stump and Simple Linear Regression remains low. In a real-time monitoring system, the variability of emissions in spring requires predictive models that can handle fluctuating environmental conditions—something ANFIS excels at. Implementing ANFIS in an online soft sensor system could allow industrial operators to maintain efficient pollutant control as environmental conditions change.

In Summer 2020 as shown in Fig. 4c, ANFIS maintains the highest performance with a high R^2^, closely followed by SMOReg and Multilayer Perceptron (MLP). For an industrial soft sensor designed for real-time PM_2.5_ monitoring, these models offer strong predictive capabilities during warmer periods. Summer might bring increased human activity, leading to higher emission levels; thus, accurate predictions are essential. Deploying models like ANFIS and SMOReg in the online dashboard can ensure prompt predictions and prevent PM_2.5_ levels from exceeding permissible limits, which is crucial for public health and regulatory compliance.

Autumn 2020 as shown in Fig. 4d shows ANFIS achieving a slightly lower R^2^ value compared to other seasons, but it still maintains a lead over other algorithms. Random Forest and M5P also exhibit improved R^2^ values, though still lower than ANFIS. Seasonal changes, such as increased use of heating systems and changes in agricultural activity, can cause fluctuations in PM_2.5_ levels. A soft sensor model embedded in an online dashboard for autumn could benefit from using ANFIS for consistency, while also incorporating Random Forest as a backup model for robustness against high-variability scenarios.

In Winter 2021 as shown in Fig. 4e, ANFIS, SMOReg, and Random Forest continue to demonstrate good predictive performance, with high R^2^ values across the board. Real-time PM_2.5_ prediction during winter is particularly challenging due to increased emissions from heating systems. In this context, an online dashboard utilizing ANFIS can provide consistently accurate predictions, allowing industrial operations to make proactive adjustments to control air quality and reduce particulate matter emissions.

During Spring 2021 as shown in Fig. 4f, ANFIS is again the dominant performer, with a notable gap between it and other models. Random Forest and Multilayer Perceptron follow but are not as consistent as ANFIS. Implementing ANFIS in a soft sensor could be critical for accurately predicting PM_2.5_ concentrations in spring when pollen and increased vehicular activity could influence air quality. Additionally, an integrated real-time dashboard could use ANFIS-based predictions to implement corrective measures, such as optimizing HVAC systems or activating pollution control equipment.

From an industrial perspective, the application of real-time PM_2.5_ prediction models, as seen in Fig. 4, could support multiple functionalities. Firstly, proactive emission management could be achieved by using ANFIS, which shows consistent accuracy across seasons. This allows industries to adjust emissions proactively and effectively, especially during critical periods when pollutant levels fluctuate significantly. Secondly, accurate prediction ensures regulatory compliance by maintaining PM_2.5_ levels within permissible limits, avoiding potential fines and supporting public health initiatives. Thirdly, the analysis highlights that a dynamic decision support system could be established, which switches models based on the time of year—for instance, prioritizing ANFIS during colder seasons and adding Random Forest for autumn to enhance robustness. Lastly, public health protection is greatly supported by the ability to predict PM_2.5_ levels accurately, minimizing the adverse impacts of poor air quality.

In conclusion, Fig. 4 provides evidence of ANFIS’s consistent performance in accurately predicting PM_2.5_ levels across various seasons, making it an excellent candidate for integration into a real-time industrial dashboard for air quality control. The seasonal variation in the performance of different models also suggests a potential benefit from a dynamic approach where the model selection is adjusted based on the time of year. Using such soft sensors can significantly enhance the decision-making capabilities of industries, ensuring regulatory compliance and reducing health impacts associated with PM_2.5_ exposure.

The analysis of all the discussed methods has revealed that the ANFIS algorithm consistently exhibits the highest accuracy, even though it is relatively more complex compared to other techniques. Meanwhile, the RF method, an ensemble approach comprising multiple trees, also demonstrated commendable performance. By combining multiple trees, RF mitigates the risk of overfitting associated with individual trees.

**Fig. 5:**
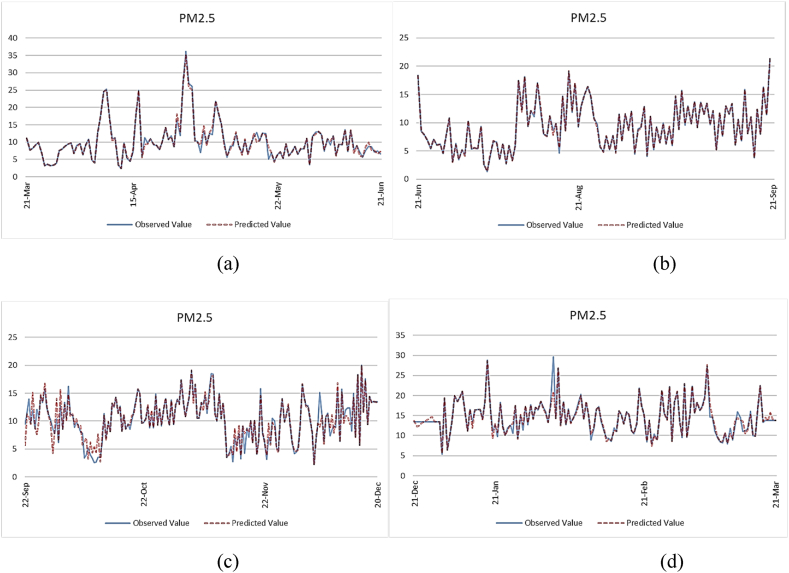
PM_2.5_ real data versus predicted data for ANFIS algorithm, a) spring 2020, b) summer 2020, c) autumn 2020, d) winter 2021.

Furthermore, we validated our data for the prediction of PM_2.5_ using the ANFIS method, as depicted in Fig. 5. The figure illustrates the predicted results of the ANFIS algorithm alongside the observed data over a one-year period. Notably, the ANFIS method showcases exceptional accuracy, effectively capturing peak values and providing managers with valuable insights for informed decision-making.

Fig. 5 shows PM_2.5_ observed versus predicted values using ANFIS for different seasons. The close overlap between observed and predicted lines indicates high prediction accuracy. In Spring 2020 (Fig. 5a) and Summer 2020 (Fig. 5b), prediction follows observed spikes well, demonstrating robustness in changing conditions. Autumn 2020 (Fig. 5c) and Winter 2021 (Fig. 5d) also exhibit minimal deviation. Industrially, this performance enables real-time PM_2.5_ monitoring for emission control, ensuring compliance and proactive adjustments to maintain air quality.

In other studies, the research could be linked to traffic analysis and climate change features incorporating artificial intelligence tools [79,80]. This would allow for the development of an online dashboard for smart cities, enabling real-time data integration and visualization, ultimately enhancing decision-making processes in urban management.

**Fig. 6:**
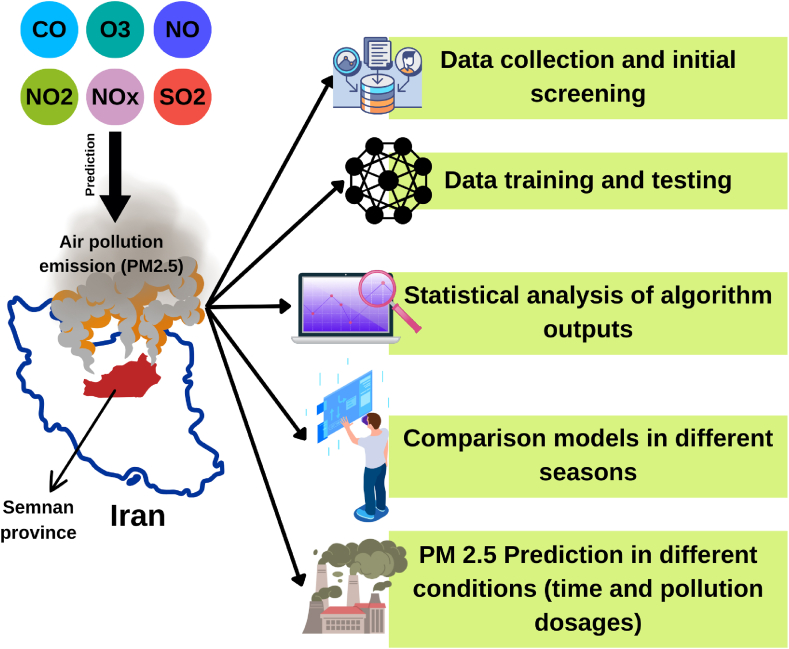
Conceptual model of the present study as a managerial insight.

With an emphasis on predictive modeling and its several phases, Fig. 6 offers a management perspective on the creation and implementation of a soft sensor for PM2.5 monitoring in smart cities. The first of the figure's many crucial elements are the identification of the various pollutants that contribute to air pollution, including CO, O₃, NO, NO₂, NOx, and SO₂. These contaminants are crucial inputs for forecasting PM2.5 emissions, a crucial indicator of air quality that has a big influence on both environmental sustainability and human health.

The "*Data Collection and Initial Screening*" step of the procedure starts with an emphasis on obtaining pollution data from Iran's Semnan Province for study. By ensuring that all required pollutant metrics are suitably assembled and preprocessed, this data collection forms the basis for the following stage.

The second phase, "*Data Training and Testing*," emphasizes how machine learning techniques are used to determine correlations between PM_2.5_ emissions and input pollution parameters. In order to use historical data trends to effectively anticipate PM_2.5_ levels under various conditions, this model training is essential. By assessing the created model's performance and pointing out any discrepancies or potential areas for development, the "*Statistical Analysis of Algorithm Outputs*" that follows guarantees the model's validity and dependability. In order to deploy the models effectively, Fig. 6 includes "*Comparison Models in Different Seasons*." By taking into consideration changes in seasonal pollutant concentrations and emission sources, this phase enables a comparison analysis across various temporal situations. The prediction model's resilience depends on this comparison and also, it is a way due to reach Sustainable Development Goals (SDGs) [[81], [82], [83]].

Finally, the "PM_2.5_ Prediction in Different Conditions" phase focuses on using the soft sensor to estimate PM2.5 levels under varying timeframes and pollution dosages. This stage provides a comprehensive decision-making tool for policymakers, allowing them to predict pollution trends and effectively mitigate air quality issues. Generally, Fig. 6 depicts a structured and systematic approach to PM_2.5_ prediction in smart cities, utilizing a machine-learning-driven soft sensor. It integrates data collection, model development, validation, and comparative seasonal analysis, aiming to ensure accurate, timely predictions to support efficient air quality management and public health initiatives.

## 4 Conclusions and future works

To sum up, this study offers a thorough examination of air quality prediction for the Iranian city of Semnan utilizing a variety of machine learning models and an ANFIS. The results show that ANFIS is the best technique for predicting pollution concentrations because it performed noticeably better than other ML models in every season. In particular, ANFIS had a R^2^ value of 0.999 in Winter 2020, with low errors as shown by an RMSE of 0.0274 and an MAE of 0.0162. These measures demonstrate ANFIS's capacity to handle non-linear correlations in air quality data and exhibit a nearly perfect fit. Comparative evaluations further illustrated those other models, such as MLP and Random Forest, exhibited moderate performance in specific instances. For example, during Summer 2020, Random Forest achieved an R^2^ value of 0.717, while MLP also showed relatively strong performance with an R^2^ value of 0.913. Nevertheless, ANFIS consistently maintained superior predictive accuracy, even when model performance dipped slightly, such as in Autumn 2020, when the R^2^ value reached 0.892. The analysis further highlighted the variability in model performance across different seasons, emphasizing the need for robust and adaptive prediction methodologies to address seasonal influences on pollutant concentrations. The study's findings highlight how important ANFIS is for predicting air quality with great reliability. ANFIS successfully captured the intricacies of pollutant interactions under various climatic situations by integrating fuzzy logic and neural network capabilities. A promising avenue for real-time air quality monitoring and decision assistance is offered by the application of ANFIS for PM₂.₅ predictions in Semnan. This is essential for reducing the harmful health effects linked to elevated pollutant levels.

Future research could focus on enhancing the robustness of predictive models by integrating deep learning algorithms or exploring hybrid approaches that combine multiple machine learning models. Additionally, incorporating metaheuristic optimization algorithms could further improve the accuracy of air quality predictions and reduce prediction errors. The use of Response Surface Methodology could also be beneficial for fine-tuning these models.

## CRediT authorship contribution statement

**Pouya Mottahedin:** Writing – original draft, Software, Methodology, Investigation, Data curation, Conceptualization. **Benyamin Chahkandi:** Writing – original draft, Visualization, Software, Methodology, Investigation, Conceptualization. **Reza Moezzi:** Writing – original draft, Visualization, Software, Methodology, Formal analysis, Data curation. **Amir M. Fathollahi-Fard:** Writing – review & editing, Writing – original draft, Visualization, Validation, Supervision, Methodology, Investigation, Conceptualization. **Mojtaba Ghandali:** Writing – original draft, Validation, Methodology, Investigation, Formal analysis. **Mohammad Gheibi:** Writing – original draft, Validation, Funding acquisition, Data curation, Conceptualization.

## Data availability

All data is available upon request from corresponding author.

## Declaration of competing interest

The authors declare the following financial interests/personal relationships which may be considered as potential competing interests: The corresponding author, Prof. Amir M. Fathollahi-Fard, is an Associate Editor in Information Science for Heliyon and was not involved in the editorial review or the decision to publish this article. If there are other authors, they declare that they have no known competing financial interests or personal relationships that could have appeared to influence the work reported in this paper.
